# Validation of a method to assess night myopia in a clinical setting

**DOI:** 10.1038/s41598-023-51062-8

**Published:** 2024-01-02

**Authors:** Andrés Gené-Sampedro, Mercedes Basulto Marset, Daniel Monsálvez Romin, Susana Montecelo Salvado, Inmaculada Bueno-Gimeno

**Affiliations:** 1 Department of Optics, and Optometry and Vision Science, University of Valencia, Facultad Física, c/ Dr. Moliner 50, 46100 Burjassot, Spain; 2 INTRAS (Research Institute on Traffic and Road Safety), University of Valencia, 46022 Valencia, Spain; 3 Department of Light and Life Vision Sciences, Research and Development EssilorLuxottica, 75012 Paris, France

**Keywords:** Translational research, Risk factors, Eye manifestations, Human behaviour

## Abstract

A study was conducted with 115 subjects who regularly drove at night to validate a refraction protocol for detecting refractive visual changes from daytime to nighttime conditions. Objective and subjective refractions were performed in both photopic and mesopic conditions, with a dark adaptation period before the mesopic subjective refraction. The results showed that in mesopic conditions, visual acuity decreased by 0.2 logMAR units on average (*p* < 0.01), and there was a myopic refractive shift of − 0.36 ± 0.20 D (*p* < 0.01). Most subjects (92.2%) exhibited a myopic refractive shift of at least 0.12 D. Compensation of refractive shift improved mesopic visual acuity by 0.06 logMAR on average (*p* < 0.01) and higher refractive shifts showed higher improvement. Night Rx was preferred by 82.1% of subjects with myopic refractive shift. Gender and age did not significantly affect the refractive shift, although myopes showed a higher shift compared to emmetropes (*p* < 0.01). The refractive shift remained stable over time when the time slot of the day did not change (*p* < 0.01). Night Rx protocol proved to be a robust and accurate method for identifying drivers with refractive changes when transitioning from photopic to mesopic conditions. The high prevalence and inter-individual variability of Rx shift highlight the need of customized refraction.

## Introduction

The human eye can detect different levels of illumination, from bright to very dim light, and the visual system can adapt its sensitivity to these conditions^1^. Interestingly, it has been observed that some subjects, under low-lighting conditions, become more myopic. The so-called “night myopia” has already been documented for more than a century and several studies have been carried out to try to give an explanation, but even today it is not fully understood and there is no single consensus about its causes^2^. To date, experimental works have tried to isolate different factors separately (Purkinje effect, eye aberrations, neural factors, adaptation to darkness), being accommodation role the one that has been suggested as the main component^3^. Furthermore, most of the mentioned experimental studies have been carried out in monocular conditions, while it has been seen that under binocular conditions this effect could be lower, probably due to fusional convergence that comes into play, stabilizing accommodation^4^. There is probably not a single cause, but a conjunction of variable contribution factors between subjects can lead to this phenomenon.

Despite all the doubts that still need to be clarified about night myopia, the manifestation of this phenomenon has recently gained clinical interest due to its impact on every-day tasks for a significant number of people, such as nighttime driving. Driving itself is a complex task that requires specific visual skills and optimal vision. In fact, we know that the visual route is what provides us with more information and the one that mainly contributes when maintaining a safe driving. Therefore, good vision is essential for safe and efficient driving^5^. Nighttime driving entails more considerable risks compared with daytime, and it involves a challenging visual environment with dim street lighting, glare from oncoming headlights, and the need to quickly adapt across a wide range of lighting levels. Certainly, fatality rates at night are up to three times higher than in the day when adjusted for driving exposure^6^. The most likely reason seems to be the reduced visibility of the nighttime road environment^2,7–9^. Night myopia significantly impairs driving performance, particularly in very bad-lit road environments^2^. Wearing corrective lenses can improve visual performance and reduce the impact of night myopia on driving performance^10^. Thus, it would be ideal for visual processing capabilities to remain in optimal conditions to perform the task over time, providing a prescription to correct night myopia.

The differences in night myopia results among studies sometimes are related to the measuring methods. Subjective refraction is still considered the gold standard for determining the optimal prescription for the correction of refractive errors, using objective methods as a baseline. However, standard subjective refractions are normally performed in a photopic viewing context which does not represent the nighttime driving environment. In this scenario, it would be more appropriate to assess the vision function under mesopic or low light conditions^11,12^. Up to the authors’ best knowledge, there is no established standard clinical procedure in such conditions.

Using standardized protocols in visual examinations can lead to more accurate diagnoses, more reliable treatment plans, and improved outcomes for patients^13–15^. A standardized protocol might help professionals to assess mesopic visual function and thus, to better address night vision needs of drivers and to provide them with custom correction. For all these reasons, this research aims to validate a protocol to perform subjective visual refractions under low lighting levels (mesopic) to identify those subjects who manifest a shift towards more myopic values with respect to their standard refraction. Also, it is desirable to estimate feasible time limits for its assessment in the clinical setting, considering the dark-adaptation time and battery of mesopic tests. Moreover, this investigation might help to understand the distribution of night myopia and the possible related individual factors contributing to its manifestation.

The purpose of this study was to validate a measurement protocol for accurate and reliable refractive nighttime prescription that can be easily implemented in practice.

## Results

The mean age of the sample was 34.51 ± 13.09 years, ranging from 18 to 63 (56% men, 44% women). Age distribution was not homogeneous, the burden of the sample consisted of 71% of non-presbyopes (18–39 years), whereas 29% were presbyopes (40–63 years). There were no significant differences in mean age according to gender (*p* > 0.05). The mean photopic and mesopic pupil size under natural conditions were 2.75 ± 0.45 mm and 5.82 ± 0.99 mm respectively for non-presbyopes and 2.39 ± 0.29 mm and 4.81 ± 0.69 mm for presbyopes. The mean spherical aberration (5-mm pupil) was − 0.00 ± 0.07 microns and 0.04 ± 0.05 microns for non-presbyopes and presbyopes respectively.

The mean (spherical equivalent) M based on objective “day” refraction was − 1.94 ± 2.30 D and on “night” refraction − 1.95 ± 2.29 D (mean change of − 0.01 ± 0.18D *p* > 0.05). In terms of ametropia distribution, 8.7% of subjects were hyperopes (M > 0.875 D), 18.3% emmetropes (− 0.625 D < M < 0.875 D), and 73.0% myopes (M < − 0.625 D). The mean manifest M based on standard (photopic) subjective refraction was − 1.93 ± 2.25 D, with a mean cylinder of − 0.76 ± 0.57 D and on night (mesopic) subjective refraction − 2.29 ± 2.28 D (mean change in sphere of − 0.36 ± 0.19 D. *p* < 0.001). The mean values of refraction (right eye) by light condition and age category are shown in Table 1. The ametropia was balanced across gender (*p* > 0.05). According to age, the presbyopic group included a higher number of hyperopic subjects, whereas non-presbyopes were more myopic (*p* < 0.001).

**Table 1:** Descriptive values of refraction of the right eye (M coefficient) and binocular visual acuity (logMAR) by light condition and age category.

	Conditions	Age	*p*	All subjects NP = 82 P = 33	Rx shift ≤ -0.12 D NP = 74 P = 32
				Mean ± SD Median (IQR)	Mean ± SD Median (IQR)
Objective Rx	Day	NP	**< *0.01***	**− 2.48 ± 1.98** − 2.25 (2.08)	**− 2.37 ± 1.92** − 2.13 (2.03)
		P		**− 0.60 ± 2.52** − 0.20 (3.57)	**− 0.68 ± 2.51** − 0.22 (3.62)
	Night	NP	**< *0.01***	**− 2.47 ± 1.99** − 2.20 (2.09)	**− 2.36 ± 1.93** − 2.05 (2.00)
		P		**− 0.67 ± 2.53** − 0.30 (3.58)	**− 0.75 ± 2.53** − 0.33 (3.54)
Subjective Std Rx	Photopic	NP	**< *0.01***	**− 2.43 ± 1.93** − 2.25 (2.10)	**− 2.32 ± 1.87** − 2.20 (2.09)
		P		**− 0.68 ± 2.52** − 0.01 (3.60)	**− 0.75 ± 2.52** − 0.19 (3.66)
Subjective Night Rx	Mesopic	NP	**< *0.01***	**− 2.74 ± 1.95** − 2.63 (2.03)	**− 2.67 ± 1.92** − 2.54 (2.04)
		P		**− 0.96 ± 2.58** − 0.25 (3.89)	**− 1.04 ± 2.58** − 0.45 (3.93)
	Mesopic w/PA	NP	**< *0.01***	**− 2.80 ± 1.95** − 2.69 (1.98)	**− 2.73 ± 1.92** − 2.67 (2.00)
		P		**− 1.02 ± 2.58** − 0.38 (3.77)	**− 1.10 ± 2.58** − 0.51 (3.92)
Subjective Rx shift	Mesopic-Photopic wo/PA	NP	> *0.05*	**− 0.31 ± 0.19** − 0.25 (0.25)	**− 0.34 ± 0.15** − 0.25 (0.25)
		P		**− 0.28 ± 0.21** − 0.25 (0.50)	**− 0.29 ± 0.21** − 0.25 (0.38)
	Mesopic-Photopic w/PA	NP	> *0.05*	**− 0.37 ± 0.20** − 0.38 (0.25)	**− 0.41 ± 0.16** − 0.38 (0.25)
		P		**− 0.34 ± 0.19** − 0.38 (0.38)	**− 0.35 ± 0.18** − 0.38 (0.31)
VA Std Rx	Photopic	NP	> *0.05*	**− 0.14 ± 0.06** − 0.14 (0.08)	**− 0.15 ± 0.06** − 0.16 (0.08)
		P		**− 0.15 ± 0.06** − 0.16 (0.08)	**− 0.15 ± 0.06** − 0.16 (0.08)
VA Std Rx	Mesopic	NP	> *0.05*	**0.06 ± 0.07** 0.06 (0.08)	**0.06 ± 0.07** 0.06 (0.08)
		P		**0.06 ± 0.07** 0.04 (0.10)	**0.06 ± 0.07** 0.04 (0.12)
VA Night Rx	Mesopic	NP	**< *0.05***	**− 0.01 ± 0.06** − 0.02 (0.10)	**− 0.02 ± 0.06** − 0.02 (0.08)
		P		**0.02 ± 0.06** 0.02 (0.10)	**0.02 ± 0.06** 0.02 (0.10)

*NP* non-presbyopes; *P* presbyopes; *PA* perceptual appreciation; Objective Rx (WAM700); Subjective Rx, subjective Rx shift and VA (VR800); *p*: p value.

Significant p-values are shown in bold italics.

**Figure 1:**
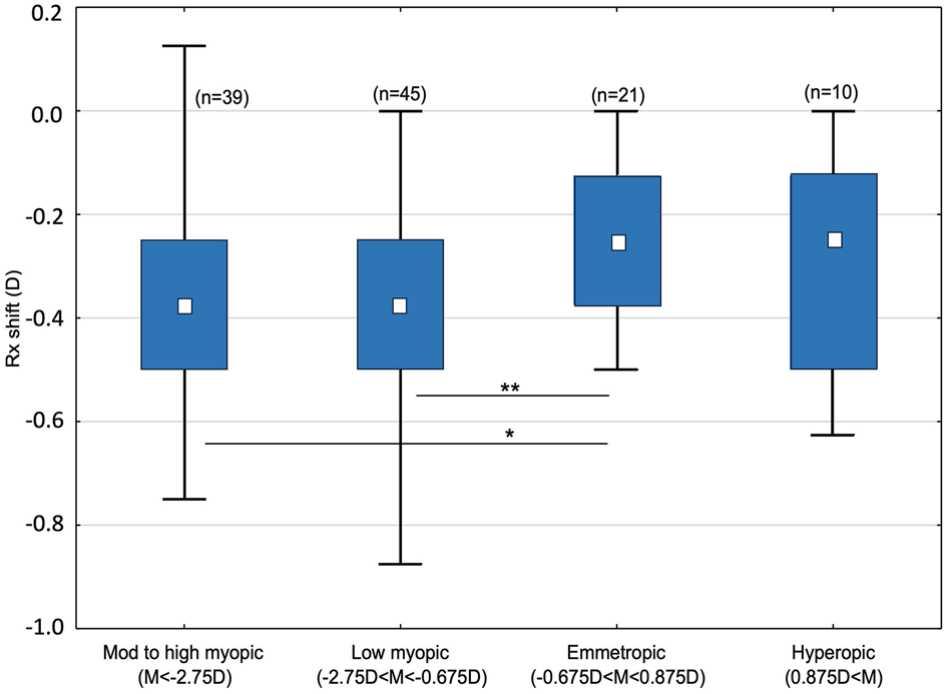
Distribution of Rx shift by ametropia category **p < 0.01 *p < 0.05.

With the PA for the calculation of the Rx shift (Rx shift w/PA), the 92.2% of the subjects (n = 106) showed a myopic shift equal or superior to − 0.12 D, 83.5% to − 0.25 D, 59.2% to − 0.37 D, 33.1% to − 0.50 D whereas the 7.8% had not myopic shift (n = 9). PA had a significant effect on Rx shift (*p* < 0.001), which was independent of the degree of gain in Night visual acuity (VA) with Night Rx (*p* < 0.001). The results showed that the Rx shift is not dependent on age or gender, neither for any age-gender category. When comparing ametropia groups, an effect on Rx shift was found. Specifically, emmetropes showed lower Rx shift than myopes (*p* < 0.05), but there was no effect of myopia degree on Rx shift (*p* > 0.05) (Fig. 1). There was no effect of astigmatism degree on Rx shift either. There was no effect of other optical factors on subjective Rx shift as mesopic pupil size (Spearman’s R = − 0.162 *p* = 0.083) or 5-mm pupil spherical aberration (valid n = 53; Spearman’s R = − 0.094 *p* > 0.05). However, spherical aberration influenced the objective Rx shift (R = − 0.957 *p* < 0.001) which showed to be less myopic than subjective Rx shift by 0.35 D in mean (*p* < 0.001).

VA decreases by 0.2 logMAR on average from photopic to mesopic conditions (*p* < 0.001). In mesopic conditions, when Rx shift is compensated VA increases by − 0.06 logMAR on average (*p* < 0.001). VA shifts vary among individuals, Standard VA shift ranges up to 0.40 logMAR and mesopic VA shift up to − 0.24 logMAR. The mean values of VA by light conditions and age category are shown in Table 1. With Night Rx, non-presbyopes show better mesopic VA (*p* = 0.006) and mesopic VA shift (*p* = 0.01) than presbyopic subjects. Values of VA shift by light conditions and age category are shown in Table 2. 100% of both non- presbyopes and presbyopes show VA loss from photopic to mesopic conditions (with Std Rx) and 89.2% of non-presbyopes and 78.1% of presbyopes had VA improvement with the Night Rx.

**Table 2:** Distribution of binocular VA shift (in letters) by light condition and age category; Subjects with Rx shift equal or superior to − 0.12 D NP non-presbyopes, P Presbyopes.

Category	Range in letters^1^	Standard VA shift^2^ (n = 106)	Standard VA shift^2^ NP (n = 74)	Standard VA shift^2^ P (n = 32)	Mesopic VA shift^3^ (n = 106)	Mesopic VA shift^3^ NP (n = 74)	Mesopic VA shift^3^ P (n = 32)
Very high gain	18 or higher	–	–	–	–	–	–
High gain	13–17	–	–	–	–	–	–
Moderate gain	8–12	–	–	–	13% (14)	18% (13)	3% (1)
Low gain	3–7	–	–	–	36% (38)	39% (29)	28% (9)
Slight gain	1–2	–	–	–	37% (39)	32% (24)	47% (15)
No change	0	–	–	–	8% (9)	7% (5)	13% (4)
Slight loss	1–2	1% (1)	1% (1)	–	6% (6)	4% (3)	9% (3)
Low loss	3–7	14% (15)	15% (11)	13% (4)	–	–	–
Moderate loss	8–12	61% (65)	62% (46)	59% (19)	–	–	–
High loss	13–17	21% (22)	19% (14)	25% (8)	–	–	–
Very high loss	18 or higher	3% (3)	3% (2)	3% (1)	–	–	–

^1^Each letter correspond to 0.02 logMAR units, ^2^VA change wearing Std Rx between mesopic and photopic conditions. ^3^VA change in mesopic conditions between Night and Std Rx.

**Figure 2:**
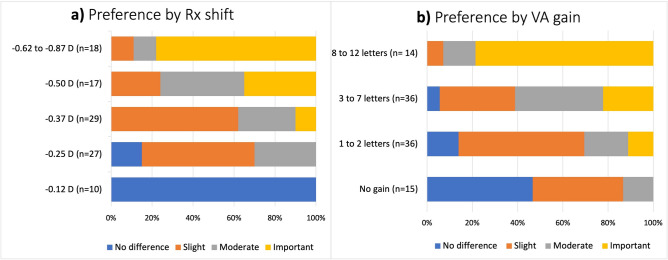
Preference difference between NIGHT and STD Rx (**a**) by Rx shift and (**b**) mesopic VA gain (in letters) when the Rx shift is compensated. Subjects with myopic shift (n = 101).

According to quality of vision preferences, for those subjects who present myopic Rx shift, Night Rx was preferred by 82.1% of subjects, 13.2% had no preference and 4.7% preferred Std Rx. Higher myopic Rx shifts led to more important subjective difference between Night Rx and Std Rx (*p* < 0.01) (Fig. 2a). However, in some cases, low Rx shifts as − 0.25 D or − 0.37 D also led to perception of important differences, whereas moderate to high Rx shifts, − 0.50 D to − 0.87 D, also yielded slight differences. On the other hand, subjects who had Rx shifts of − 0.12 D did not perceive any difference. In terms of VA the higher the gain when Rx shift is compensated, the more important the difference perceived between Night and Std Rx (Fig. 2b).

**Figure 3:**
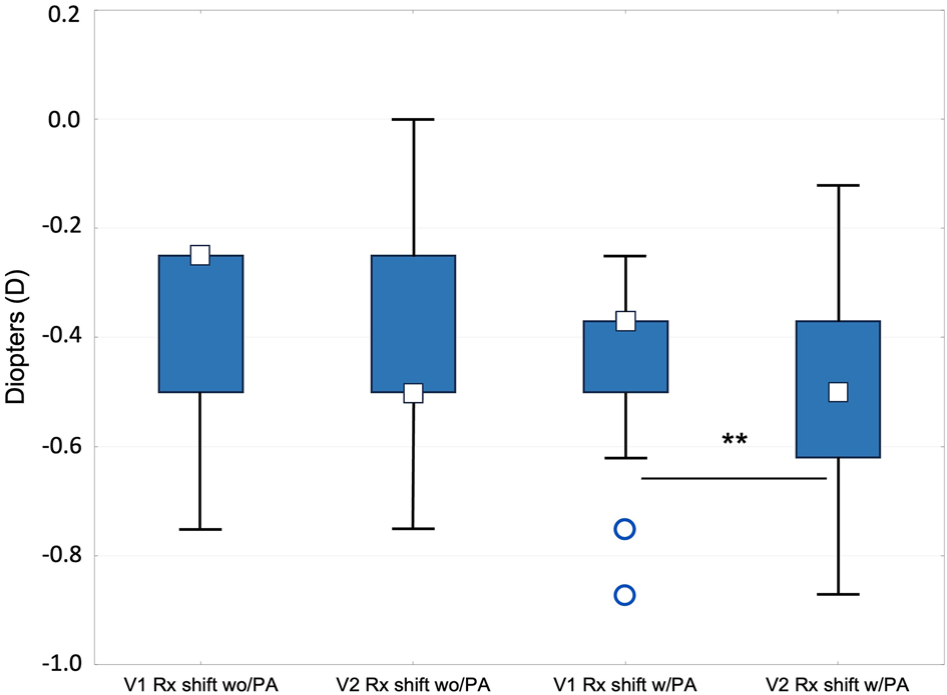
Inter-session reproducibility of the refractive shift without (Rx shift wo/PA) and with perceptual appreciation (Rx shift w/PA). V1 (first visit) and V2 (second visit). **p < 0.01.

The assessment of the reproducibility of the proposed method (n = 54, mean age 27.1 ± 6.2 years) showed no significant differences in either M or Rx shift between visit 1 (V1) and visit 2 (V2) without PA (wo/PA). However, taking the PA into account, the Rx shift was more myopic in V2 (*p* < 0.01) (Fig. 3), although this difference was still close to zero (− 0.07 D).

**Figure 4:**
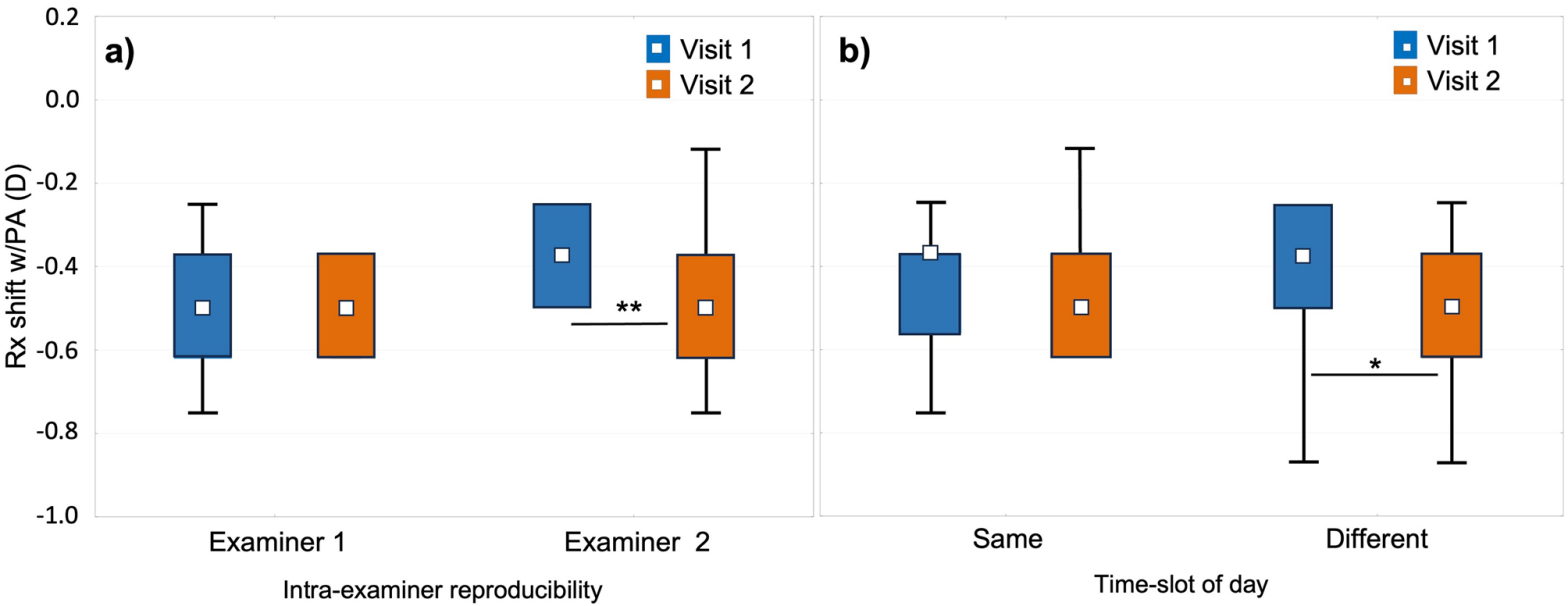
Reproducibility of refractive shift with perceptual appreciation (Rx shift w/PA) (**a**) Intra-examiner effect (**b**) Effect of time slot of the day. V1 (first visit) and V2 (second visit) **p < 0.01, *p < 0.05.

Differences of refractive and VA parameters as well as LoA between visits are shown in Table 3. The reproducibility of Rx shift w/PA was near 60% within ± 0.12 D and 94% within ± 0.25 D. The number of days elapsed between V1 and V2 [< 60 days (n = 28) and > 60 days (n = 26)] had no effect on Rx shift wo/PA. The Rx shift w/PA was more myopic when elapsed time was greater than 60 days (mean difference − 0.08 D) (*p* = 0.047). There was found an examiner effect. The intra-examiner effect (same examiner between visits) yielded more myopic Rx shift values (w/PA) in V2 for examiner 2 (− 0.12 D in average, *p* < 0.01), but not for examiner 1 (*p* = 0.60) (Fig. 4a). The inter-examiner (different examiners between visits) did not significantly change this Rx shift. Lastly, the reproducibility of the Rx shift was influenced by the differences in the time of the day at which the protocol was performed. When time of day did not change between visits (within ± 3 h), there was found no difference in Rx shift (w/PA) independently of the number of days between visits or whether the examiner changes or not (*p* > 0.05). When time of day changed between visits (more than ± 3 h), the Rx shift (w/PA) was more myopic in V2 (mean difference − 0.09 D, *p* < 0.05) (Fig. 4b).

**Table 3:** Inter-session reproducibility of refractive coefficients M, J0 and J45 of the right eye (diopters) and binocular visual acuity (logMAR) by light condition.

Parameter (n = 54)	Light Condition	Visit 1 Mean ± SD	Visit 2 Mean ± SD	LoA	*p*
Subjective Std Rx—M	Photopic	− 2.12 ± 1.62	− 2.12 ± 1.67	0.46	0.951
Subjective Std Rx – J0	Photopic	0.05 ± 0.38	0.04 ± 0.36	0.20	0.668
Subjective Std Rx – J45	Photopic	0.00 ± 0.17	− 0.02 ± 0.18	0.18	0.106
Subjective Night Rx -M	Mesopic	− 2.49 ± 1.67	− 2.52 ± 1.66	0.43	0.278
Subjective Rx shift wo/PA	Mesopic-Photopic	− 0.37 ± 0.15	− 0.40 ± 0.15	0.31	0.135
Subjective Rx shift w/PA	Mesopic-Photopic	− 0.43 ± 0.15	− 0.50 ± 0.15	0.31	***0.006***
VA Std Rx	Photopic	− 0.15 ± 0.06	− 0.15 ± 0.05	0.11	0.779
VA Std Rx	Mesopic	0.06 ± 0.06	0.07 ± 0.07	0.15	0.331
VA Night Rx	Mesopic	− 0.02 ± 0.05	− 0.02 ± 0.06	0.10	0.238
Std VA shift	Mesopic-Photopic(Std Rx)	0.21 ± 0.06	0.22 ± 0.06	0.13	0.297
Mesopic VA shift	Mesopic (Night-Std Rx)	− 0.08 ± 0.06	− 0.09 ± 0.05	0.14	0.839

Std Rx (Standard subjective refraction); Night Rx (Mesopic subjective refraction); Rx shift (Refractive shift); woPA (without perceptual appreciation); wPA (with perceptual appreciation); VA Std Rx (binocular visual acuity wearing Std Rx); VA Night Rx (binocular visual acuity wearing Night Rx); Std VA shift (visual acuity shift from mesopic to photopic conditions, wearing standard refraction); Mesopic VA shift (VA shift in mesopic conditions between Night and Std Rx); LoA: 95% Limits of Agreement.

*p*: p-value.

Significant values are in bold italics.

## Discussion

In this study, we developed and tested a new protocol to measure mesopic subjective refraction (Night Rx protocol). The use of protocols in the visual examination of refractive errors is important to ensure accurate measurement and correction of refractive errors^13,16,17^. By following established protocols, clinicians can ensure that all aspects of the examination are performed consistently and thoroughly, reducing the risk of errors or oversights, leading to improved visual outcomes for patients. Validating protocols is essential for optimizing refractive prescription accuracy^16,18,19^.

Subjects in this research were dark-adapted to a unique short period (5 min). In mesopic vision, both cones and rods photoreceptors contribute to the visual response and both foveal and peripheral vision are involved. It is a complex intermediate vision state as the degree of cones and rods interaction is constantly changing due to the eye’s adaptation to a dynamic range of luminance levels and different visual stimuli. In mesopic vision, when the light level decreases, cones increase their sensitivity by reduction of the spatial resolution whereas rods are highly sensitive but slower in terms of adaptation and response^20^. In the dark, cones adapt fast by increasing its sensitivity until a maximum threshold after 5–10 min and rods (which are saturated) slowly start adapting over time. Other studies have also shown a maximum improvement in mesopic VA after 6 min of dark adaptation^21^. Most of the studies assessed mesopic visual function under a high range of dark-adaptation times from 5 to 20 min^21,22^. Since we aim to implement this protocol in a clinical setting, it is desirable to optimize the duration of the measurements to avoid fatigue. For this reason and based on previous findings, a dark-adaptation of 5 min was conducted prior to mesopic refraction^21,22^.

We corroborated that VA measures deteriorated in mesopic conditions compared to photopic conditions. While driving at night, visibility of targets decreases, which increases visual processing time and the risk of accidents, even with small reductions^9,23,24^. Myopic Rx shifts measured by the night Rx protocol in this study varied from − 0.12 D to − 0.87 D (− 0.36 D on average). Several researchers have suggested that optical correction of night myopia would only have a marginal benefit for VA^25,26^. However, Hessler et al.^27^ reported that the optical compensation of this refractive shift improves the visual function under mesopic conditions. Thus, optimizing night driving performances might improve road safety. The influence of undercorrected refractive error as low as − 0.50 D impacts night-driving performances as reading road signs^28^ or pedestrian recognition^29^. Quality of vision and comfort also improves with optimized correction for mesopic levels^27,30^. In our study, whereas Rx shift of − 0.12 D showed no benefit on perceived quality of vision on simulated real-life scene, changes as low as − 0.25 D led to significant improvement in individual’s perception (Fig. 2a). Further research is necessary to assess the clinical relevance of Rx shift magnitude in night driving activity.

In this study, myopic subjects had more night myopia compared to emmetropic and hyperopic ones. Atchison et al.^31^ found that people with myopia had reduced visual performance under low-light conditions, including reduced VA and contrast sensitivity. Other studies showed that night myopia values of − 0.75 D or higher affect night-driving performance and increase number of accidents^8^. After reviewing our findings, myopic subjective Rx shift (measured with Night Rx protocol and ETDRS chart) is not reflected by objective Rx measurements (WAM700) which is significant less myopic by − 0.35 D, most likely due to spatial configuration of visual stimuli and other instrument related factors. Furthermore, optical factors as pupil size and 5 mm-pupil spherical aberration do not have influence in subjective Rx shift under low-light conditions, at least in healthy phakic patients. Influence of pupil-related factors as spherical aberration on night vision has been reported previously^2,4^. As light environment decreases pupil size increases and effect of excess of positive spherical aberration on visual performance is greatest. Subjective refractive error becomes more myopic for some stimulus configuration typical of night driving^32^. Previous research shown that spherical and chromatic aberrations are unlikely to affect night myopia^3^. Hessler et al.^27^ did not found relationship between subjective luminance-dependent refraction and pupil size-dependent objective refraction^24^. In our study, spherical aberration does influence the objective Rx shift but not the subjective change. Thus, subjective Night Rx and thus, the mesopic Rx shift should be influenced by other factors than optical characteristics of the eye such as neural factors—receptoral (retina) and post-receptoral complex visual processing-linked to the change in light environment^25,26^.

The participants in our study were healthy. However, it is crucial to understand the importance of various optical considerations, especially when there are disturbances in the ocular media. The level of disturbances tends to rise as the pupils dilate, which can lead to a heightened intraocular scattering effect Moreover, straylight increases with age even in normal eyes^33^. This effect can potentially impact various visual functions, including contrast sensitivity and disability glare. It is noteworthy that this impact is not limited to individuals with compromised ocular health but can also affect those with seemingly healthy eyes and excellent visual acuity^34^.

As in previous studies^35^, in our research, presbyopes and non-presbyopes presented different physiological characteristics; presbyopes showed to be less myopic or more hyperopic, the pupil size decreases, and positive spherical aberration (5-mm pupil) increases compared to non-presbyopes. However, these physiological differences are not reflected in Rx shift compared to non presbyopes. Change in refraction at low-light levels has been related to accommodation errors more than to spherical aberration or pupil size^3,36^. In our study, physiological decrease of accommodation with age does not impact measured subjective Rx shift when comparing non-presbyopic and presbyopic populations. One of the key aspects of vision in driving is VA, which refers to the sharpness of vision. Good VA allows drivers to see clearly and quickly identify important information, such as road signs and other vehicles. Sivak and Schoettle^37^ showed that VA was a significant predictor of night driving performance, and that older drivers with poorer VA had more difficulty driving at night. Poor VA, on the other hand, can make it difficult for drivers to see important details, which can increase the risk of accidents. Owsley et al.^38^ showed that drivers with poorer VA were more likely to have a crash, and that the risk of a crash increased as VA decreased. The risk of accidents increased significantly as VA decreased^39^. Besides VA, other visual functions playing an important role on night driving such as contrast sensitivity^11^, disability glare^12^ and halo perception^12,40,41^ should be evaluated in order to characterize the role of vision in night driving performances.

For our research, we have chosen mesopic VA as a benchmark. High-contrast, photopic VA has been shown to be a poor predictor of closed-road night-time driving^11^, with mesopic VA being a better predictor of night-time driving than standard photopic measures^11,12^. However, there are limitations to measurements of mesopic VA, which can be unreliable due to variations in chart luminance at these low light levels^42,43^. Previous studies demonstrated repeatability of mesopic VA at 0.75 cd/m^2^^44,45^ or about 2.0 log units lower than the photopic test^44^. However, measures at 0.75 cd/m^2^ are clinically more significant than those at higher mesopic luminance levels as 3 cd/m^2^^44^. Our results showed similar reproducibility limits for 1.1 cd/m^2^ (0.10 95% LoA for Night VA). Moreover, nighttime road lighting standards are between 0.3 and 2.0 cd/m^2^^46^. For all these reasons, mesopic luminance of 1.1 cd/m^2^ as an intermediate value of lighting standards and inferior by 2.0 log units than photopic level (181 cd/m^2^), seems to be an adequate first choice for mesopic VA assessment for both age categories. Photopic VA chart luminance levels in this research follows standards for subjective refraction^47^ (ISO NORM 10938:2016) usually conducted on clinical practice. Nevertheless, higher levels of VA chart for determining Std Rx which are not evaluated in this study would give more insight on shift in refraction from photopic to mesopic conditions.

Our results agree with those of the study by Johnson^25^, who measured VA under several different low luminance levels using sinusoidal gratings. Even with night myopia correction, VA was significantly decreased in mesopic conditions compared to photopic conditions. This result strongly suggests that factors other than refraction influence VA under low luminance conditions. It has been suggested that decreases in VA occur at lower light levels because of neural factors associated with decreasing retinal illuminance, more than optical blur from myopic shifts^22,25,26^. Our results further support this idea, changing light environment degrades binocular VA by 2 lines on average independently of the subject’s Rx shift. Moreover, Rx shift compensation leads to a VA gain of 1 line on average which increases with the Rx shift, myopic Rx shifts higher than − 0.50 D improve by twice the VA in mesopic conditions compared to lower myopic Rx shifts (− 0.25 to − 0.37 D). Population shows a high inter-individual variability in VA shifts, a loss up to 4 VA lines due to change in light environment and a gain with the Night Rx up to 2 VA lines. Both age groups show VA improvement with Night Rx. However, difference in mesopic VA with Night Rx is of 2 letters lower for presbyopes. Other factors as gender do not influence VA gain when Rx shift is compensated.

Quality of vision at nighttime depends not only on VA, but PA needs to be considered. In line with previous research in photopic conditions on dioptric power sensitivity^16^, our results show that PA adjustment does influence subjective refraction by improving visual perception and comfort compared to the MPMVA endpoint in mesopic conditions. Subjects were able to benefit of PA adjustment which is reflected by a Rx shift refining in more precise steps (0.12 D) than the clinical standards (0.25 D). PA impacts Rx shift and improves quality of vision independently of VA gain produced by the Rx shift compensation and other physiological factors as age, gender, or ametropia. Benefit of Night Rx is appreciated subjectively. For those subjects who present myopic Rx shift, Night Rx is preferred over Std Rx (82.1%), and for half of the subjects, the difference was seen as moderate to important. Subjective benefit increases for higher myopic Rx shifts or higher mesopic VA gains. However, the high inter-individual variability in the perceived benefit evidences a variability in inter-individual mesopic sensitivity. Low myopic Rx shift and low mesopic VA gain due to Rx shift compensation may also lead to a high subjective benefit and vice versa, high changes in visual performances may be perceived as slighter benefit. The high inter-individual variability of the Rx shift compensation evidence differences in subject’s sensitivity in mesopic conditions. Thus, it is desirable to custom mesopic subjective refraction according to individual’s specific needs.

From our data, we have observed that refraction coefficients in photopic conditions are stable over time; Mean differences between visits were close to zero and 95% LoA are within the expected limits based on previous studies assessing repeatability of conventional subjective Rx, from ± 0.48 D and ± 0.63 D for the M (SE) coefficient^48^ and from ± 0.16 D to ± 0.50 D in astigmatic coefficients. Therefore, we can conclude that Std Rx is reproducible and no significant biological evolution of subject ametropia occurred.

In our understanding there is no previous research on reproducibility of subjective refraction in mesopic conditions. The results of this study are in line with those in the literature for photopic conditions^48^. Rosenfield and Chiu^49^ who assessed repeatability of subjective refraction under ideal conditions such as a single masked examiner, same timeslot of day and 5 repeated times, showed 95% LoA of ± 0.29 D of M suggesting that a change of ± 0.50 D should be considered as clinically significant. Results suggest that, when using a guided standardized protocol in a controlled environment, the reproducibility of mesopic subjective refraction is good and comparable to previous studies of Rx in photopic conditions, and differences are clinically and statistically not relevant in optimized experimental conditions as same examiner and same moment of the day.

Although all the procedures were carefully designed and conducted, there are several limitations and future research lines to mention. First, it would have been more informative to have additional age categories for validating any distinct results or age-related concerns that may arise among this demographic. However, due to the insufficient sample size the subjects were analyzed in two main groups based on their presbyopic status, which were representative for statistical analysis. Second, reproducibility has been assessed in non-presbyopic population due to potential physiological variations in younger eyes are lower and presbyopic sample was more limited varying in age categories. Assessment of reproducibility in older subjects should be considered to gain a more comprehensive understanding of effect of night myopia in this specific population. Third, majority of the population in this investigation is myope. It would have been of interest to extend night myopia assessment to higher number of hyperopes and emmetropes for more insight of ametropia effect. Fourth, according to our research, measuring and comparing VA and perceived visual quality with on real-life night driving driver scene can help evaluate night myopia. Since there may be multiple causes for night myopia, it is recommended to include additional visual performance metrics like contrast sensitivity, stereo acuity, and glare halos for a thorough assessment of how it affects night driving. Fifth, moreover, small variation of chart background luminance and dark adaptation undertaken period should be investigated due to the potential influence on mesopic refraction and associated impact on VA and perceived quality of vision.

We used specific refraction instrument to perform 0.01 D precise refraction and adapted screen to attain and control mesopic luminance level of visual stimuli. However, for clinicians who lack access to specific instrumentation, the protocol can still be implemented using traditional optometric setups such as a manual phoropter or trial frame with 0.25/0.12 D steps. While it may not result in an exact night refraction, it is possible to perform. Mesopic VA can be measured without the need for mesopic-vision dedicated screens. However, it is crucial to regulate the background luminance of the VA chart screen as well as the ambient lighting^21^. If a VA chart projector is being used, neutral density filters^21,44^ or two crossed linear polarizing sheets can be utilized to decrease the luminance to the desired mesopic conditions. In this latter case, in some parts of the refractive or sensory examination, it is not recommended to perform tests that require additional polarized lenses to avoid affecting the test results. For instance, when adjusting biocular balance refraction, instead of using polarized lenses to dissociate the vision of each eye, it is recommended to use vertical prisms. This approach ensures accurate results without any interference from the lenses.

92.2% of subjects in this study showed different correction between photopic and mesopic vision. When considering the use of different visual corrections for night and day vision, it's important to carefully weigh the advantages and practical challenges. One key benefit is the potential for improved night vision. In low light conditions and with increased glare, visual issues like refractive errors can be worsened^12,27,28^. Specialized corrections for night vision can help people see more clearly in these situations and reduce visual disturbances caused by headlights and streetlights, making driving at night safer. Additionally, wearing separate corrections for nighttime activities can enhance overall comfort, especially for those with specific visual needs as nighttime driving. One of the challenges of using separate glasses for day and night vision is the added cost and inconvenience, it can be difficult to remember to bring the appropriate pair or keep track of both. Moreover, not everyone may need this approach, as it primarily benefits individuals with specific night vision difficulties or requirements. A case-by-case decision should be made based on individual visual needs and preferences, with consultation from an eye care professional to determine suitability. For instance, those subjects with a night myopia of − 0.12 D did not report any difference between using it or not. Thus, it is suggested that individuals with night myopia ≤ − 0.25 D should correct their distance vision for activities in low-light conditions (see Fig. 2).

Finally, although the visual task analysis in this manuscript is considered from the point of view of night-time driving, visual tasks are relevant also in other mesopic applications such as marine, aviation, security lighting, and the safe movement and visual orientation of pedestrians in urban areas as well as bad weather conditions.

## Conclusion

Nighttime driving poses unique challenges to drivers, and vision also plays a crucial role in ensuring safety on the road. A standardized protocol to conduct mesopic refraction may help eye care professionals to address night vision complaints related to VA loss during night driving. Results prove the accuracy and feasibility of the Night Rx protocol to identify drivers who presented a different Rx between photopic and mesopic conditions. Night Rx and Rx shift are reproducible over time and compensation of Rx shift leads to a significant VA gain and subjective benefit in terms of quality of vision. Conducting a 5-min dark-adaptation, the Night Rx protocol is optimized in time and feasible for clinical setting. There is a high prevalence of the Rx shift and a high inter-individual variability, thus the need of customized measurement. In conclusion, drivers with night myopia should consider wearing corrective lenses to improve their visual performance in nighttime driving and reduce the risk of accidents on the road.

## Methods

### Sample

This study included 115 subjects (82 non-presbyopes and 33 presbyopes). Inclusion criteria were age (between 18 and 65 years old), best-corrected distance visual acuity (BCDVA) of + 0.10 logMAR or better, distance spherical equivalent between − 8.00 and + 6.00 diopters (D), cylindrical error up to − 3.00 D, anisometropia up to 1.50 D, and driving at night regularly (at least 4 days per week). Exclusion criteria were any previous ocular surgery, pathology, untreated and/or uncontrolled systemic condition, any medical treatment or medication which might have an influence on vision or interfere with study assessments. Subjects with migraine, epilepsy, binocular vision problems, permanent wearing of contact lenses, or tinted lenses and with knowledge in optometry, ophthalmic lenses or optics were also excluded from the study.

### Experimental design

This was a monocentric, exploratory, comparative, and controlled study performed at the University of Valencia and approved by the Ethics Committee of Research in Humans of the Ethics Commission in Experimental Research of University of Valencia and adhered to the tenets of the Declaration of Helsinki. All subjects were informed of the study characteristics and protocols, and informed consent form was obtained.

### Procedure

Subjects were given a general health questionnaire for the inclusion criteria. The same experienced examiner/s (A.G-S. and M.B-M) performed all the measurements. A preliminary ocular examination including BCDVA, binocular screening, stereo acuity, and ocular motility test were carried out to assess inclusion requirements. Objective refraction, ocular aberrometry and pupillometry were performed with Wave Analyzer Medica 700 (WAM700), (Essilor, France). This multifunctional eye diagnostic device^16^ provides in one single measurement two monocular refraction values for photopic (or “Day”) and mesopic (or “Night”) conditions, giving both low and high order aberrations at 3-mm and 5-mm pupil apertures. It uses Hartmann-Shack aberrometer. The stimulus luminance was 4 cd/m^2^ (Konica Minolta LS-110 luminance meter). For each subject, a single measurement was conducted in a dark room to ensure sufficient pupil width. Objective refractions were recorded in both eyes and pupil size and spherical aberration were recorded only in the right eye because data from both eyes is similar in a healthy population^50^. The subjective refraction was evaluated with the Vision-R^TM^800 (VR-800) automated phoropter (Essilor, France) which allows resolution of 0.01 D steps by continuous lens power variation^16^. Two subjective refractions (photopic and mesopic) were performed at 6.0 m under controlled light environment following 3 steps. Firstly, under photopic conditions (lightroom 45 lx), secondly, a dark adaption under scotopic conditions (dark room < 0.01 lx), and thirdly, under mesopic conditions (lightroom 0.01 lx). Spectral irradiance measurements were done at eye level using a Konica Minolta CL-200 Chroma Meter illuminance spectrophotometer. Randomized ETDRS charts were used to avoid the learning effect in the VA measurement. These charts were presented on the Vision-C 600 (VC600) screen (Essilor, France), at two different background luminance: photopic level 181 cd/m^2^ (standard refraction) and mesopic level 1.1 cd/m^2^ (night refraction) and letter contrast (Weber fraction) higher than 90%. In order to attain desired mesopic luminance level, the screen backlight was decreased and ETDRS charts were adapted with a grey background. Luminance measurements were done using a ColorCAL MKII Colorimeter. In Supplementary Fig. S1 online shows a flowchart with the four steps of the refraction protocol. In photopic conditions, objective refraction was used as starting point for the standard subjective refraction. Monocular fogging/defogging and cross-cylinder technique, biocular and binocular balance were performed^16^. The most positive refraction that provided the best visual acuity (MPMVA) on ETDRS chart was considered for both monocular and binocular endpoints. Subject was then dark-adapted for 5 min (VR800 refractor head’s occluders on and room light off < 0.01 lx). In mesopic conditions, a fogging of + 0.50 D was applied while subject was looking at the ETDRS chart (mesopic). Then, the MPMVA was obtained by adjusting the binocular sphere from the photopic MPMVA value maintaining the astigmatic component unchanged. The endpoint of the mesopic refraction was the perceptual appreciation (PA) which is a method to “refine” the refraction value to give visual comfort to the subject. An algorithm based on the PA test adjusts the mesopic binocular MPMVA in 0.12 D steps (see Supplementary Fig. S2 online). Subjects compared the binocular MPMVA with addition of − 0.25 D sphere and graded the difference in slight/moderate or important. Finally, subjects were able to compare photopic and mesopic refractions on a simulated real-life night-driving scene displayed on the VC600 under mesopic conditions and graded their quality of vision preference as slight, moderate, or important.

To validate the protocol, reproducibility was tested on a group of subjects from the study (n = 54). After an average of 60 days following the initial visit, refractive tests were repeated under the same experimental conditions. The results of refraction, the spherical equivalent (M coefficient) and refraction shift (Rx shift), and VA were compared between the first and second visits.

The metrics derived from each examination are defined as follows:

- **Photopic or standard refraction (Std Rx)**: standard binocular refraction in photopic conditions.
- **Mesopic or night refraction (Night Rx)**: binocular refraction in mesopic conditions.
- **Refractive shift (Rx shift)**: difference in binocular sphere between the night (mesopic) refraction and the standard (photopic) refraction. It is usually myopic.
- **Photopic visual acuity (in logMAR units)**: binocular VA in photopic conditions wearing the Std Rx.
- **Mesopic visual acuity (in logMAR units)**: binocular VA in mesopic conditions wearing the Night Rx.
- **Standard visual acuity shift (in logMAR units)**: change in binocular VA between mesopic and photopic lighting conditions wearing the Std Rx.
- **Mesopic visual acuity shift (in logMAR units)**: change in binocular VA in mesopic conditions between the Night Rx and Std Rx. It is the change in VA due to compensation of the Rx shift.

### Statistical analysis

It was performed using the SPSS software for macOS (version 28.0, IBM Corp., USA) and the Statistica software for PC (version 14.0.1.25 TIBCO Inc USA) and it was analysed in descriptive terms. Parametric or non-parametric tests were used according to sample size and/or normality data. The normality was checked using the Kolmogorov–Smirnov test. For those paired variables that did not follow a normal distribution, the Wilcoxon sign test was used. For the repeated measures, the non-parametric Friedman test was used, equivalent to the analysis of variance for variables that do not meet the normality condition. For bi-variants independent samples the Mann–Whitney test was used and the Kruskal–Wallis for more than two variables.

This study analyzed both objective and subjective refractive data for each eye individually. Pairwise Wilcoxon tests were conducted to check for differences between the eyes. Since no significant differences were found, this manuscript reports only the analysis of refractive data from the right eye.

The level of significance was defined as *p* < 0.05. Reproducibility analysis was used to quantify the contribution of the number of days elapsed between visits, examiner, and time of the day on refraction components. Limits of agreement (LoA) were estimated by ± 1.96 standard deviation.

### Supplementary Information

Supplementary Figures.

## Data Availability

The datasets used and/or analysed during the current study will are available from the corresponding author on reasonable request.
