# Publisher Correction: Validation of a method to assess night myopia in a clinical setting

**DOI:** 10.1038/s41598-024-52881-z

**Published:** 2024-01-30

**Authors:** Andrés Gené-Sampedro, Mercedes Basulto Marset, Daniel Monsálvez Romin, Susana Montecelo Salvado, Inmaculada Bueno-Gimeno

**Affiliations:** 1 Department of Optics, and Optometry and Vision Science, University of Valencia, Facultad Física, c/ Dr. Moliner 50, 46100 Burjassot, Spain; 2 INTRAS (Research Institute on Traffic and Road Safety), University of Valencia, 46022 Valencia, Spain; 3 Department of Light and Life Vision Sciences, Research and Development EssilorLuxottica, 75012 Paris, France

Correction to: *Scientific Reports* 10.1038/s41598-023-51062-8, published online 02 January 2024

The original version of this Article contained an error, where the names of the authors Andrés Gené-Sampedro, Mercedes Basulto Marset, Daniel Monsálvez Romin, Susana Montecelo Salvado and Inmaculada Bueno-Gimeno were incorrectly given as Gené-Sampedro Andrés, Basulto Marset Mercedes, Monsálvez Romin Daniel, Montecelo Salvado Susana and Bueno-Gimeno Inmaculada.

The original Article has been corrected.

