# Supplementary material for: Validation of a method to assess night myopia in a clinical setting
Source: Sci Rep. 2024 Jan 2;14:293. doi: 10.1038/s41598-023-51062-8 (PMC10762171; doi:10.1038/s41598-023-51062-8)
Supplement: Supplementary file 1 — Supplementary Figures. [file 41598_2023_51062_MOESM1_ESM.pdf]

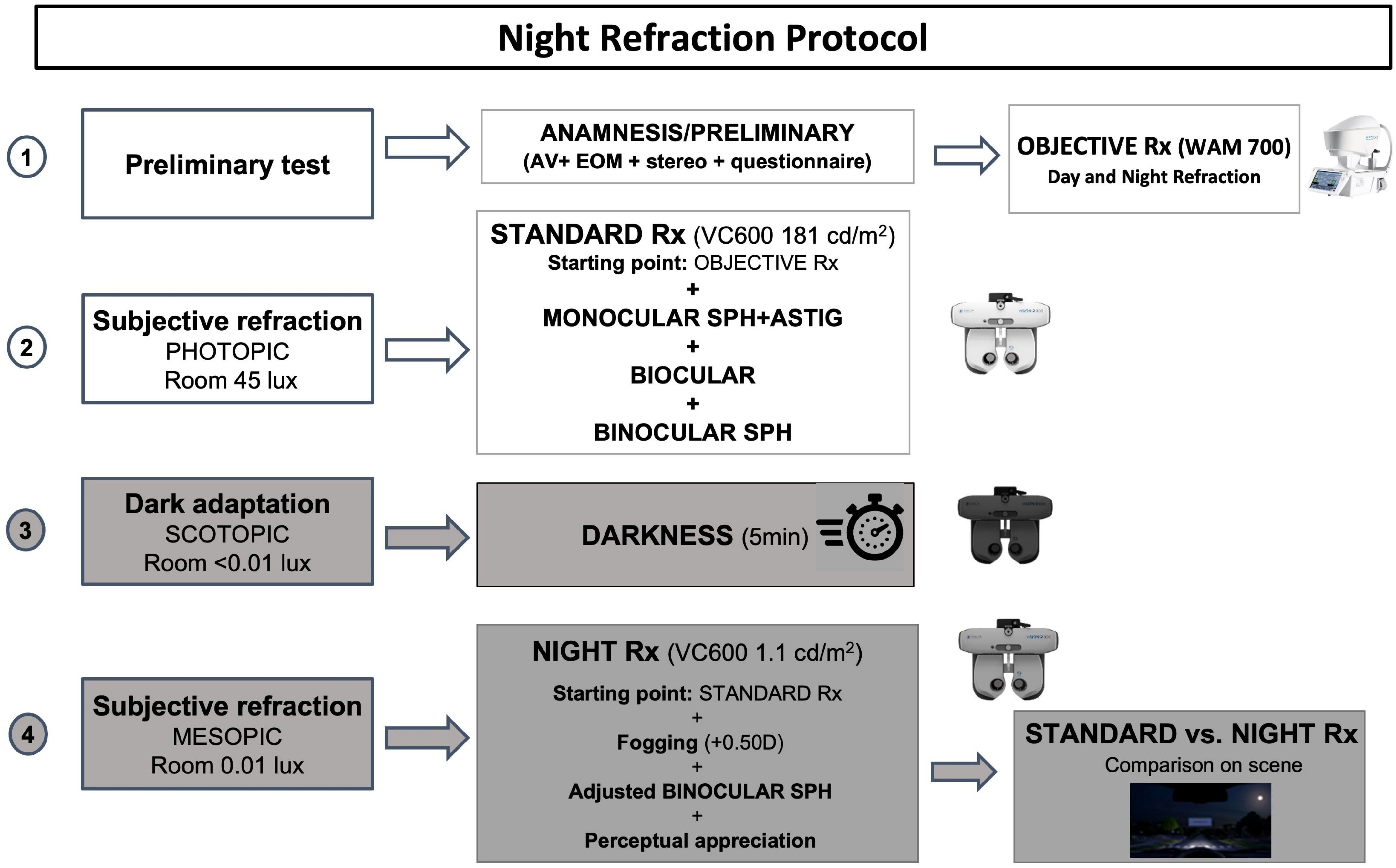

Supplementary S1.- Night refraction protocol flowchart.

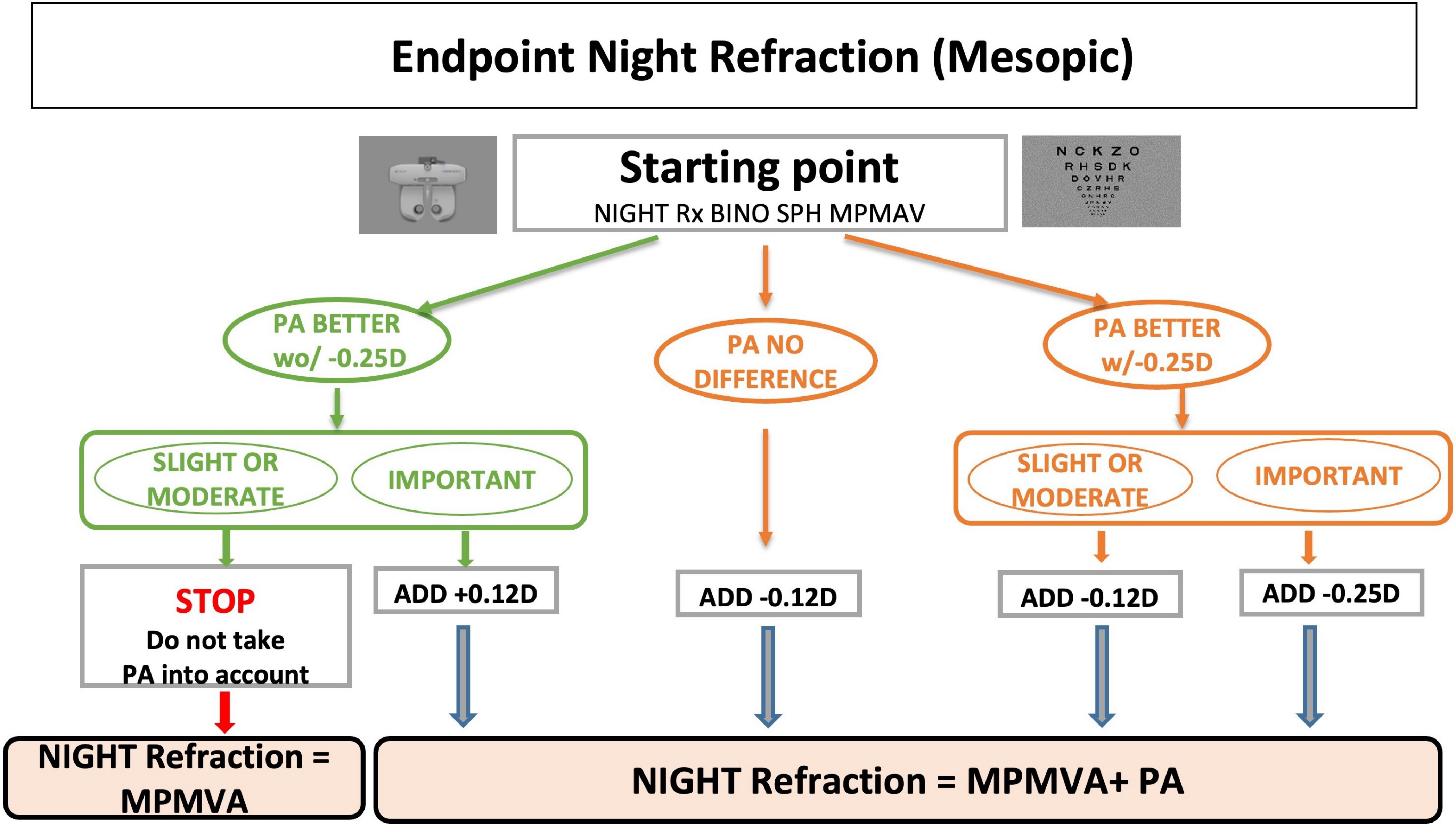

PA (Perceptual Appreciation); **MPMVA** (Maximum Positive Maximum Visual Acuity); **NIGHT Rx** (Night Subjective Refraction)

Supplementary S2.- Night refraction perceptual algorithm (endpoint).
